# Attentive and Pre-Attentive Processes in Change Detection and Identification

**DOI:** 10.1371/journal.pone.0042851

**Published:** 2012-08-16

**Authors:** Howard C. Hughes, Gideon Paul Caplovitz, Rebecca A. Loucks, Robert Fendrich

**Affiliations:** 1 Department of Psychological and Brain Sciences, Dartmouth College, Hanover, New Hampshire, United States of America; 2 Department of Psychology, University of Nevada Reno, Reno, Nevada, United States of America; CNRS - Université Claude Bernard Lyon 1, France

## Abstract

In studies of change blindness, observers often have the phenomenological impression that the blindness is overcome all at once, so that change detection, localization and identification apparently occur together. Three experiments are described that explore dissociations between these processes using a discrete trial procedure in which 2 visual frames are presented sequentially with no intervening inter-frame-interval. The results reveal that change detection and localization are essentially perfect under these conditions regardless of the number of elements in the display, which is consistent with the idea that change detection and localization are mediated by pre-attentive parallel processes.

In contrast, identification accuracy for an item before it changes is generally poor, and is heavily dependent on the number of items displayed. Identification accuracy after a change is substantially better, but depends on the new item's duration. This suggests that the change captures attention, which substantially enhances the likelihood of correctly identifying the new item. However, the results also reveal a limited capacity to identify unattended items. Specifically, we provide evidence that strongly suggests that, at least under these conditions, observers were able to identify two items without focused attention. Our results further suggest that spatial pre-cues that attract attention to an item before the change occurs simply ensure that the cued item is one of the two whose identity is encoded.

## Introduction

Under many circumstances, the visual system is exquisitely sensitive to a change in the visual scene. This sensitivity allows for the rapid detection and identification of important objects and events, i.e., those that have suddenly appeared, disappeared or begun to move. This paper explores the relationships between the detection, localization and identification of objects that have changed. Specifically, we investigate whether the same processes mediate change detection and change identification, or whether one can occur in the absence of the other.

Visual attention plays a central role in most accounts of change detection and identification [1]–[4]. When two scenes are presented in immediate succession (i.e., with a 0 ms inter-stimulus interval, ISI), changes between them are often effortlessly detected, and are said to ‘pop out’ [5], [6]. Most accounts attribute this ‘pop out’ to low-level massively parallel mechanisms whose activation automatically draws attention to an isolated transient [7]–[9]. This shift of attention is normally accompanied by a ‘reflexive’ shift of gaze to the new event, unless endogenous control signals keep the eyes fixed (e.g., [10]).

Under certain circumstances, however, changes can go undetected by these ‘early warning’ pre-attentive mechanisms. This can occur if a change occurs very slowly over time [11], or if many things change at the same time (e.g., [6], [12]). Conditions such as these either fail to activate (slow changes) or overwhelm (multiple changes) the change detection system and lead to a state of ‘change blindness’ that can only be overcome by a serial search of the scene until attention (and the eyes) happens to land at the critical location (e.g., [2], [6], [12]–[14]). Interestingly attending to the location of change, in and of itself, is not sufficient to overcome change blindness, since changes often remain undetected when an observer's gaze is directed right at the critical location the moment that the change occurs [15], [16].

However, the relationship between the *detection* of a change and the *identification* of what has actually changed remains to be clarified [3], [17], [18]. These processes are easily conflated. The ‘flicker paradigm’, a widely used method for producing change blindness [6], illustrates how this conflation can occur. In this paradigm two nearly identical images are presented in an alternating sequence separated by a brief intervening interval. The change between the two images may be the deletion, displacement or some other alteration (change in the color or shape) of an object within the scene. Detecting a change that occurs under these conditions often requires a prolonged period of visual search. This is likely because the global transients produced by the blank interval overwhelm the pre-attentive change detection system [12]. Often the search process is conceptualized in terms of a series of shifts of attention coupled (if the task permits) with shifts of gaze [6], [13], [14], [16]. When the change is finally detected, it is often accompanied by an ‘aha’ moment in which observers suddenly notice the critical object switching from state A to state B, and produces the subjective impression that detection and identification occurred simultaneously.

This impression of simultaneous change detection and identification is consistent with theories that propose that, in change blindness paradigms, change detection depends upon a comparison between a current visual input and one stored in visual short-term memory (VSTM). According to these theories, changes are detected in these paradigms when a mismatch between these two representations is detected [2], [6]. If we assume that 1) change detection requires a mismatch between a current visual representation and one stored in VSTM, and 2) only attended items are stored in VSTM, then this general account postulates that the changing item must be attended to (and thus identified) *before* the change is detected. Thus, detecting the mismatch also entails identifying what has changed. This may be the case when observers are attempting to overcome change *blindness*, but there is also ample evidence that motion and other visual primitives can be detected and segmented in parallel by processes that precede attention (e.g., [19]–[21]). Thus, it is important to not over-extrapolate from the conditions that produce change blindness to those that more generally mediate change detection. To some extent, the study of the phenomenon of change blindness has confused consideration of the role of attention in change detection and identification, so that the terms ‘change detection’ and ‘change identification’ have been used in an inconsistent (and even interchangeable) manner. Because the detection and identification are often phenomenologically closely coupled in studies that employ the flicker paradigm, the term ‘change detection’ has often been used to describe what is actually change detection and identification (e.g., [6], [13], [14], [22]). For example, in a 2002 review by Rensink [2] the term change detection is used (p. 246) to denote “… not only detection proper… but also identification (reporting what the change is) and localization (reporting where the change is).” Later the same paper (p. 257) acknowledges that “detection is not *necessarily* identical to localization or identification” (italics added), and, in fact, Rensink notes near the end of the review that the difference between change detection and identification remains an “open issue” that needs to be addressed (p.269).

Several subsequent reports have indicated that change detection and identification are not necessarily coincident, because observers can sometimes accurately detect a change without being able to identify what has changed [1], [3], [23], [24]. Turatto and Bridgeman demonstrated this dissociation between change detection and change identification using a ‘one shot’ paradigm in which the change only occurred once on each trial and there were no irrelevant transients [3]. These authors examined the effects of biasing attention on the accuracy of both change detection and identification. The method involved using color cues to indicate which items in a multi-element display would be most likely to undergo either a shape change or a deletion. The assumption was that subjects would use these color cues to prioritize the deployment of their attention during the first frame of the two-frame sequence. The results indicated that the identification of shape changes was substantially facilitated for items that had been prioritized by the color cues, while there was significant but relatively minor effect of cuing on the latency and accuracy of detection *per se*. These results indicate that detection and identification can be dissociated. A related finding showed that when a target in an array changes its luminance or *both* its luminance *and* color, the luminance change is readily detected and localized, but observers do not know whether a color change has also occurred [24]. On the other hand, another report using natural scenes reported similar (and sometimes equivalent) detection and identification rates [18]. Thus, the literature is equivocal regarding the degree to which change detection and identification are separable.

Interestingly, Turatto and Bridgeman [3] only examined shape changes under these ‘single shot’ exposure conditions, while color changes were the only type of change that showed equivalent detection and identification rates in the report by Mondy and Coltheart [18]. Shape changes can produce spatiotemporal transients that mimic motion (e.g., [25]), and motion is among the many visual features that can be detected pre-attentively [20], [26]. It would therefore be valuable to determine whether change detection and identification are dissociable for changes that are probably unrelated to mechanisms of motion detection.

In view of these theoretical conflations and empirical confusions concerning the relationship between change detection and change identification, we thought it would be useful to address this distinction in more detail. Because we wished to explore the information available when conditions favor the detection of changes rather than illuminate the nature of change blindness, we followed the logic of Turatto and Bridgeman [3] and studied the information available when the two displays were presented with no intervening blank interval. This allowed us to explore the role played by attention in the detection of visual change and the identification of what had actually changed when these two perceptual functions are dissociated.

The first experiment examined the information gleaned from an array of 18 items (colored shapes) displayed at equal eccentricities from fixation. On most trials, one item changed its color, shape or both color and shape. Observers reported 1) whether they had detected any change, and if so, 2) its location, 3) the identity of the item before the change, and 4) the identity of the new (post-change) item. We presumed that subjects performed this task using their ambient vision to monitor information from the entire array rather than focused attention, since they 1) were required to fixate the center of the array, and 2) had no information about which item would undergo a change and therefore no incentive to covertly direct their attention to any particular display element. While one could construe this monitoring process as a diffusion of attention over an extended spatial area, if this area is large this still amounts to monitoring ambient visual information. Our results show that under these conditions changes detection and localization are essentially perfect. The identification accuracies for the pre-change item, and to a lesser extent the post-change item, were much less accurate. Thus, the detection and localization of a change in either color or shape is readily dissociated from the ability to identify what had changed. This result suggests detection and localization are mediated by a parallel omnibus change detecting system that receives input from both the magnocellular and parvocelluar pathways. In addition, the results support the views that item identifications are facilitated by attention, and that the transients produced by a change draw attention to that location. The data also suggest that information about the type of change (color, shape, or both) which has occurred may be available even when the exact identities of the changed items cannot be specified, and that color and shape changes have a comparable potency with respect to their ability to capture attention.

The second experiment examined a similar task in which the ‘critical item’ was deleted rather than being replaced with a new item, and also varied the number of items displayed. Once again, detection and localization of this item were essentially perfect, and clearly exceeded identification accuracy. However, identification accuracy was substantially improved over that found in Experiment 1. This can be attributed to the capture of attention by the offset, followed by attentive processing of the persisting icon of the deleted item. However, in addition to the improvement provided by attention to the icon, there was a pronounced improvement in performance as the number of items displayed was decreased. Since attention to the icon should not have been affected by the number of items displayed, this implies the identities of some items were encoded before the critical item disappeared. The results of Experiment 2 therefore not only confirm that the detection of a change and the identification of what has changed are readily dissociated but also imply that attention may not be completely essential for items to be identified. Experiment 3 explored this implication.

The third experiment addressed the question of whether attention is *necessary* for the identification and placement of an item into VSTM by employing spatial pre-cues to direct attention to the most likely location of the change. The results indicated that attention to a particular item virtually insured that its pre-change identity would be encoded, but a small number of unattended items were also stored in VSTM.

## Experiment 1

### Methods

#### Participants

The observers were two of the authors (GPC and HCH) and seven naïve Dartmouth undergraduate students (3 males, 4 females). All observers had normal or corrected to normal vision.

#### Ethics Statement

All the experimental procedures received the approval of the Internal Review Board of Dartmouth College, and all observers affirmed their willingness to participate by signing an informed consent document. Undergraduate participants received an experiment credit that could be applied to their Introduction to Psychology course.

#### Stimuli and procedures

Stimuli were generated using the FreeBASIC programming language and viewed binocularly on a 17″ CRT monitor (refresh rate = 60 Hz) from a distance of 57 cm. Each display consisted of a circular array of 18 equally spaced elements. The array had a radius of 8° of visual angle and was centered on fixation. Each element was one of four shapes (diamond, circle, triangle or cross) and had one of four possible colors (red, green, blue, yellow), yielding 16 possible color-shape combinations. The elements were equated in physical luminance (35.0 cd/m2), subtended 1.5° of visual angle, and were presented against a dark grey (2.3 cd/m^2^) background. The color and shape of each element in the array was determined randomly with two constraints: Each of the 16 possible elements had to appear in at least one location, and the identical color or shape could not appear in more than 3 contiguous positions.

Observers initiated each trial with a mouse click. The initial stimulus array (Frame 1) was presented for 500 milliseconds (ms), and was immediately replaced (0 ms inter-stimulus interval, ISI) by a second array (Frame 2). The duration of this second array (the Frame 2 duration) was either 50 ms, 100 ms, or 500 ms. The items in Frame 2 were identical to those in Frame 1 except that one of the Frame 1 elements (the ‘target’ or ‘critical item’) changed color, shape, or both color and shape. There were also catch trials in which none of the items changed. Fifteen hundred ms after the onset of Frame 1 a ‘report location’ display appeared. This consisted of a ring of 18 1.5° diameter circles that appeared in the same locations as the Frame 1 and 2 elements, along with a central 1.5° circle. Observers indicated the location of the change by moving a cursor over the circle at that location and clicking the left mouse button. If no change was detected the observer clicked the central circle.

**Figure 1:**
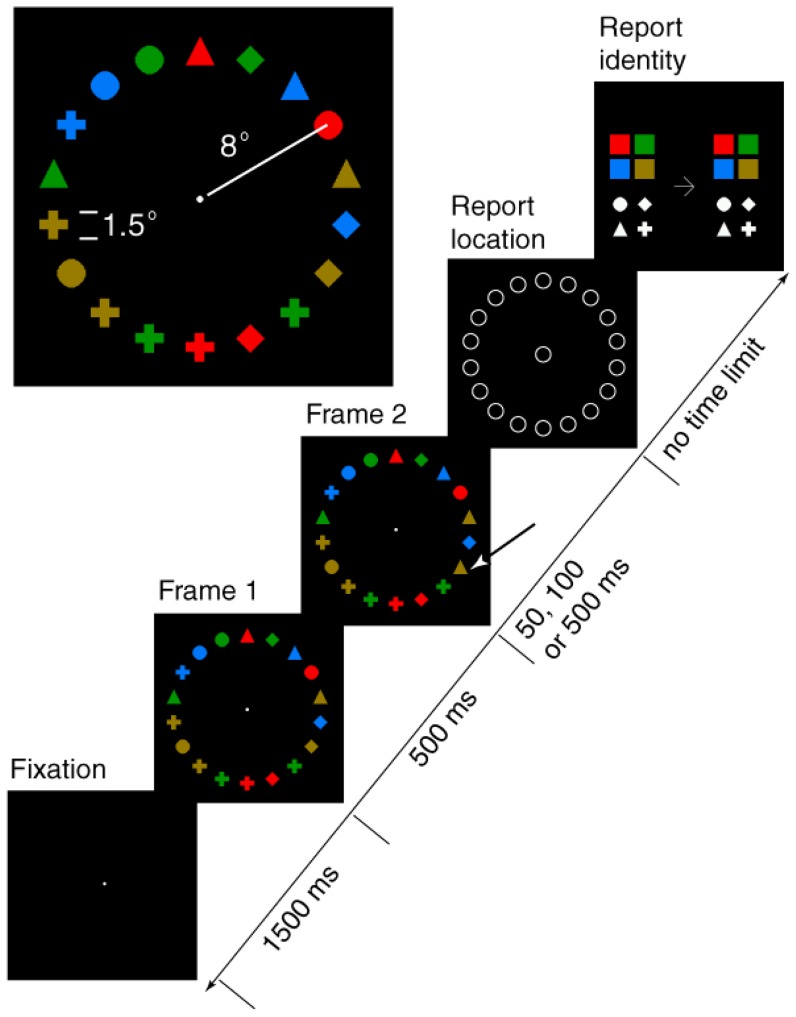
The sequence and stimuli used in **Experiment 1.** The dimensions of the stimuli and their eccentricities are illustrated in the upper left panel. The time line indicates the sequence of events within a trial, beginning with the appearance of the fixation point and ending with the report identity display. The location of the changed item is indicated by the arrow in Frame 2 (yellow diamond to yellow triangle).

If no change was reported the fixation mark reappeared on an otherwise blank screen and after 1500 ms the observer could initiate the next trial. If a change was reported in any location, that location was entered into a data file and the ‘report location’ display was replaced with a ‘report ID’ display. This display is also shown in Figure 1. It consisted of two sets of shape exemplars and two sets of colored exemplars. Using the left mouse button, observers clicked on the appropriate shape and color exemplars on the left side of the display to indicate the shape and color of the Frame 1 element that had changed, and clicked on the exemplars on the right side of the display to indicate the shape and color of the item that had replaced it in Frame 2. Once selected, the exemplars were marked by a dark outline so that observers could review their choices. Observers could make their selections in any order and could correct their selections. When the final selection had been made, observers signaled their selections were complete with a right mouse click. This caused the central fixation spot to reappear, and after 1500 ms the next trial could be initiated.

Trials were pseudo-randomly ordered such that an equal number (8) of shape, color and combined color-shape changes occurred for each of the three Frame 2 durations. In addition, there were 8 no-change catch-trials for each of these three durations. Thus, a given experimental session consisted of a total of 96 trials. There were nine observers: five participated in six experimental sessions, and four participated in four sessions.

#### Eyetracking

Vertical and horizontal positions of the right eye were digitized at 240 Hz using a SensoMotoric Instruments (SMI) Hi-Speed eye-tracking system combined with custom Labview software. Eye position was calibrated using a five-point cross-shaped calibration grid, with points at eccentricities of 8° from a central point on the horizontal and vertical meridians. Observers were instructed to maintain central fixation while the Frame 1 and Frame 2 displays were on the screen. They were free to look wherever they wished after Frame 2 disappeared. Observers' eye positions were monitored in real-time and digitally recorded for off-line analysis. Trials in which fixation deviated by more than 2.0° from the fixation point during Frames 1 and 2 were excluded from the analyses.

### Results

The entire data set consisted of 4416 trials, 91.2% (4027) of which were deemed usable. Only 1.5% of the trials were rejected due to fixation losses. The remaining rejections were due to noisy or incomplete eye records (tracker loss or eye blinks).

#### Change Detection and Localization Accuracy

Changes were detected 93.1% of the time (89.3% with the short Frame 2 duration, 96.0% with the medium Frame 2 duration, and 94.0% with the long Frame 2 duration). The false alarm rate was less than 0.4% and did not vary as a function of Frame 2 duration. For four of the nine participants, there were no false alarms. When the change was detected, observers correctly reported its position an average of 97.7% of the time (97.5% for the short, 97.3% for the medium, and 98.4% for the long Frame 2 duration). Moreover, in every case in which there was a localization error, the location indicated was adjacent to the correct location.

Detection rates can be examined as a function of ‘change type’. Let us designate a color change as *CC*, a shape change as SC, and both a color and shape change as *BC*. We found that detection rates depended on the type of change (CC = 80.7% , SC = 99.0% and BC = 99.7%, *F*(2,16) = 103, p<0.0001). Post-hoc pair-wise comparisons (t-tests) indicated that the detection rate for CC was significantly lower than either SC or BC (both p<0.001). The small 0.7% difference between SC and BC was not significant (p = 0.062).

#### Change Identification Accuracy

The accuracy with which participants could identify the items that had changed was very much lower than the detection and localization accuracy. When changes were detected, participants were able to correctly report the identity of the target item in both Frame 1 and Frame 2 on only 14.6% of the trials. To a large extent, their errors were due to failures to correctly identify the Frame 1 element, which was successfully identified on only 18.5% of the trials. However, this identification rate is significantly (*t*(8) = 6.97, *p*<0.001) better than the rate of 6.66% (1/15) expected by chance. This 1/15 value assumes that the identity of the Frame 2 item was known and is eliminated from consideration because a *change* was being reported. Not surprisingly, Frame 2 duration had little effect on Frame 1 identification accuracy (19%, 19% and 18% for the short, medium, and long durations respectively). Identification performance was much better for the Frame 2 elements; these were correctly identified on 77.9% of the trials. In contrast to the Frame 1 identification rate, Frame 2 accuracy rates improved as the duration of Frame 2 increased (63.1%, 79.7%, and 90.8% for the short, medium, and long durations respectively, statistics provided below).

**Figure 2:**
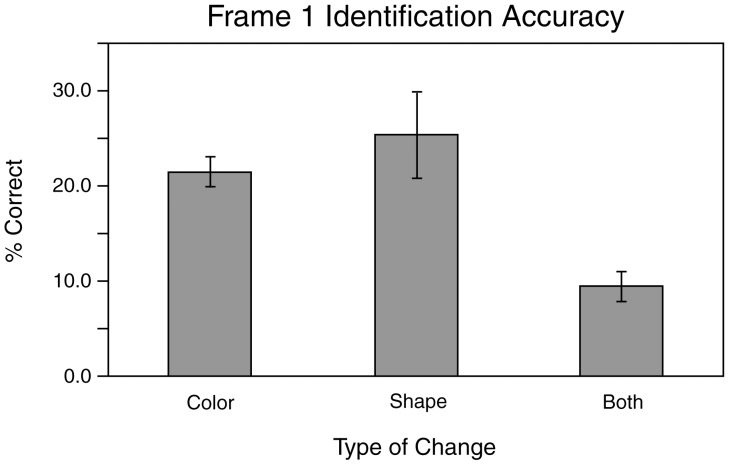
Frame 1 identification accuracy in **Experiment 1.** The percent correct identifications, averaged across subjects, are illustrated for color changes, shape changes or combined color and shape changes. Error bars indicate standard errors of the mean (+/−1 SEM).

**Figure 3:**
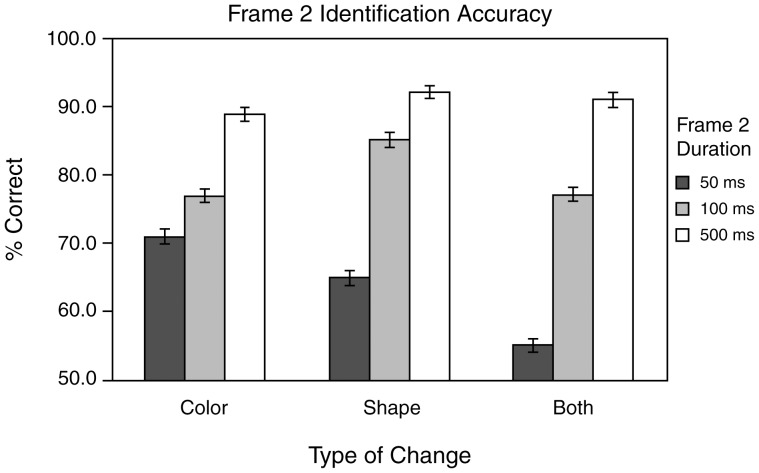
Frame 2 identification accuracy in **Experiment 1.** The percent correct identifications, averaged across subjects, are illustrated for each of the 3 Frame 2 durations (50, 100 and 500 ms) and each type of change (color, shape or color and shape). Error bars indicate +/−1 SEM.

Identification accuracy can also be examined as a function of change type. As can be seen in Figure 2, Frame 1 identification rates were conspicuously lower with the BC change type than with the CC and SC change types (BC = 9.5%, CC = 21.2%, SC = 25.5%). Two 3×3 two-way repeated measures ANOVAs (one for accuracy rates for Frame 1 and one for Frame 2) were used to statistically quantify the main effects of Frame 2 duration (three levels: short, medium and long) and change type (three levels: CC,SC and BC) on identification accuracy. The analysis of Frame 1 accuracy revealed a significant main effect of change type (*F*(2,16) = 7.71, *p*<0.005) and confirmed that Frame 2 duration had no significant effect on Frame 1 identification rates: main effect of Frame 2 duration *F*(2,16) = 0.203, *p*>0.1, interaction (*F*(4,32) = 0.873, *p*>0.49). Thus, only the type of change influenced Frame 1 identification. Post-hoc 2-tail paired t-tests comparing the mean performance of the three change types support the view that the effect of change type is due to the poorer performance in the BC trials. The BC accuracy rate is significantly lower than the CC (*t*(8) = 4.41, *p*<0.005) and SC rates (*t*(8) = 3.32, *p*<0.02), but the CC and SC rates do not differ from each other (*t*(8) = 0.40, *p*>0.4). However, the Frame 2 ANOVA indicates that both the main effects of duration (*F*(2,16) = 87.9, *p*<0.0001) and change type are significant (*F*(2,16) = 9.7, *p*<0.002), and these factors interact (*F*(4,32) = 5.36, *p*<0.02). Figure 3 show the effects of duration and change type on Frame 2 accuracy rates. As can be seen in Figure 3, increased Frame 2 durations led to increased Frame 2 identification rates for all three types of change. Post-hoc paired t-tests reveal that, collapsed across change type, this improvement is significant both when the short and medium durations are compared (*t*(8) = 10.42, *p*<0.0001) and when the medium and long durations are compared (*t*(8) = 4.49, *p*<0.002). The effects of change type are less obvious, but appear to be attributable to poorer performance in the BC condition when the duration is short. As was the case for Frame 1, paired t-tests performed on the Frame 2 accuracy rates for each change type collapsed across duration indicate that the BC accuracy rate is significantly lower than both the CC (*p*<0.02) and SC (*p*<0.001) rates, which do not differ from each other (*t*(8) = 0.94. *p*>0.1). The fact that the reduced accuracy in the BC condition occurs only with the short Frame 2 duration can account for the change type by duration interaction.

#### Information about the type of change

One way to explain the overall above chance accuracy of Frame 1 identification would be to posit that observers had some information regarding the *type of change*, even if they did not know the identity of the critical item. If, for instance, an observer knew a color change had occurred and they knew the identity of the new item in Frame 2, they could restrict their guess to the 3 remaining colored items that had the new item's shape. Even if this partial information was only available on some subset of the trials, it could support above chance accuracy in the absence of specific information about the color of the target item before the change. We tested these speculations by focusing on trials in which the Frame 1 element was *incorrectly* identified and determined whether there was any tendency for the *type of change* to be reported correctly even though the wrong element was chosen.

Identification errors were sorted into 9 groups: CC-CC, CC-SC, CC-BC, SC-CC, SC-SC, SC-BC, BC-CC, BC-SC and BC-BC. The first pair of letters in these group labels indicates the actual change type, and the second pair signifies the change type that was reported. The reported change was determined by examining the relationship between the responses for Frame 1 and Frame 2. For example, if the observer reported that a green diamond turned into a blue diamond, this would be recorded as a reported color change. In contrast, if the observer reported a green diamond turned into a green triangle, this would be recorded as a shape change. The results of this tabulation are presented in Table 1, with columns showing the actual change type and rows indicating the reported change type.

**Table 1:** This table provides a comparison of the actual changes with the reported change types (pooled across 9 observers) when the critical item in Frame 1 was misidentified.

	Actual		
Reported	CC	SC	BC
CC	0.51 (364)	0.13 (103)	0.15 (141)
SC	0.18 (126)	0.4 (317)	0.4 (377)
BC	0.31 (217)	0.47 (373)	0.45 (424)
Total Trials	707	794	942

Values in the cells show the proportion of the total trials in each column that each change type was reported. Numbers in parentheses show the number of trials these proportions represent. Assume that an observer correctly reports that a change has occurred and is able to correctly identify the Frame 2 item. There are then 14 elements that can be chosen for Frame 1 that will lead to an incorrect response, because of the 16 possible choices, one corresponds to a correct response and another corresponds to no change having occurred. If the actual change is a *color-only* change, only two of these 14 choices will result in the observer correctly specifying a color-only change despite having incorrectly identified the Frame 1 element. This is because a color-only change requires that the Frame 1 and Frame 2 *shapes* be identical. As an illustration, if the actual change is a switch from a blue diamond to a green diamond, to designate a color-only change (while still incorrectly identifying Frame 1), the observer must select either a yellow or red diamond. If the observer's choice of the Frame 1 item is a random guess between these two, the odds of correctly specifying there has been a color change will therefore be 0.143 (two out of 14). Corresponding logic dictates that if a shape-only change has actually occurred, the odds of the observer correctly specifying that there has been a shape-only change will also be 0.143 (two out of 14), while if both the color and shape have changed, the chance odds that the observer will specify that both have changed will be 0.571 (eight out of 14). Of course, the accuracy rate for correctly identifying the new item in Frame 2 was not perfect – the average across all conditions was 78.9%. We ran a Monte Carlo simulation to determine what effect this imperfect Frame 2 performance would have on the likelihood of randomly choosing the correct type of change. In this simulation the Frame 2 item was correctly identified on 79% of the trials and picked randomly on the remaining 21%. In both cases, the Frame 1 choice was picked at random with the constraint that it could not be either the correct Frame 1 item or the chosen Frame 2 item. The simulation showed that, for color and shape changes, including the incorrect Frame 2 choices increased the odds of picking the correct change type to 0.156. Including the incorrect Frame 2 choices on both-change trials increased the probability of correctly guessing the change type to 0.578. The outcomes in Table 1 were evaluated with one sample 2-tailed t-tests that compared the proportions observed for the 9 participants with these expected values. The tests indicated the observed proportion of correct change type choices was significantly greater than predicted by chance when there was a color change (*t*(8) = 6.34, *p*<0.001) or a shape change (*t*(8) = 3.36, *p*<0.01). In accord with this observation, all nine observers were more likely to report a color than shape change when there was a color-only change, and more likely to report a shape than color change when there was a shape-only change. In contrast, when the actual change was a combined color-shape change, the proportion of ‘both’ responses was actually *lower* than the expected chance value, although the difference was non-significant (*t*(8) = −1.43, ns). The data suggest this occurred because a combined color-shape change was as likely to be reported as a “shape-change” as a “both.”

These results suggest that in trials in which only one feature changed, observers were sometimes able to successfully access and use information about the type of change when attempting to identify the Frame 1 element. This hypothesis makes a prediction that can be explicitly tested: observers' ability to select the *correct* Frame 1 item should depend on a correct identification of the Frame 2 item. This is because information about the nature of the change can only be useful if the Frame 2 item is known. We tested this prediction by comparing the conditional probability of correctly identifying the Frame 1 item depending upon whether the Frame 2 item was correctly identified. Those conditional probabilities were submitted to a 2×3 repeated-measures ANOVA with factors Frame 2 correctness (correct or incorrect) and Frame 2 duration (short, medium or long). The analysis revealed a significant main effect of Frame 2 correctness (Frame 1 identification was more accurate when Frame 2 was correct [19.6%[ than when Frame 2 was incorrect [13.6%], *F*(1,8) = 6.69, *p*<0.04), but neither the main effect of Frame 2 duration (*F*(2,16) = 0.099, *p*>0.90) nor the interaction between correctness and duration (*F*(2,16) = 1.85, *p*<0.19) was significant.

It therefore appears that observers had some (albeit imperfect) information regarding the *type* of change that had occurred even when the Frame 1 item could not be correctly specified. However, this information was only advantageous when the change was restricted to a single attribute (the color or shape). When an item's shape and color changed together, the shape-change signal appears to have dominated, producing confusion between shape changes and shape *plus* color changes.

### Discussion: Experiment 1

The principle point made by Experiment 1 is that the detection and localization of a single abrupt change in a complex visual display are readily dissociable from accurate identification of what actually changed. The very substantial differences between detection and identification accuracy imply that different cognitive operations subserve each process [3], [17], [23]. A second point is that while motion related signals may contribute to pre-attentive change detection, the detection process operates almost as efficiently for color changes as it does for changes in shape. Shape changes necessarily entail changes in the positions of contours, and thus might be expected to activate magnocellular pathways that have long been associated with the capture of attention [27], [28]. In contrast, color changes do not involve any motion or change in the position of contours, and presumably depend more on responses in color-coded parvocellular pathways. Both mechanisms appear to provide input to an omnibus ‘change detector’, and the conclusion that both types of change are detected by massively parallel low-level detectors is consistent with the large literature on parallel processing of simple features in visual search tasks (e.g., [19], [21]).

The observed confusion between color+shape changes and shape only changes is reminiscent of an observation that color changes are not detected when they are accompanied by a luminance increment [24]. It appears that shape changes and/or luminance increments can mask color changes, but an isolated color change captures attention almost as efficiently as the shifting contours associated with a change in shape.

#### Change Detection and Localization as parallel pre-attentive processes

The very high accuracy rates for detection and localization are consistent with the premise that change detection and localization occur without a need for spatial attention. Rather, change detection appears to be mediated by an array of parallel channels capable of localizing spatiotemporal transients over a large portion of the visual field [7], [9], [27]. Activation of these ‘change detectors’ seems to control rather than depend upon visual spatial attention [8], [29]; but see [30].

#### The Facilitation of Identification by Attention

In contrast to detection and localization, and in accord with previous investigations (e.g., [31], [32]), we found change identification appears to dramatically benefit from the alignment of spatial attention with the location of the change. This dependence of identification on attention can explain the pronounced difference in identification accuracy for items presented before and after a visual change (i.e., Frame 1 identification accuracy was much poorer than Frame 2 identification accuracy). The identity of an item occupying the critical location before the change occurs is unlikely to be encoded because the number of items presented greatly exceeds the span of apprehension (e.g., [33]). If however, a spatiotemporal transient is detected pre-attentively and causes a shift of attention to that location, then that shift of attention can enable encoding of the new item's identity. Because it takes time to move attention [34] this process is dependent on the exposure duration of the new item. The idea that changes reflexively attract attention can explain the increase in Frame 2 identification accuracy relative to Frame 1. It can also explain the finding that post-change identification accuracy declines with decreases in Frame 2 duration (Figure 3), since the shorter the Frame 2 duration, the less time there is available to shift attention to that item before it disappears. The increased difficulty in correctly identifying the post change item given a combined color and shape change, coupled with the reduced opportunity for attentional processing of that item when Frame 2 durations are short, appears to account for the interaction of these two factors that is evident in Figure 3.

We also consider it noteworthy that the capture of attention appeared to be nearly as effective with our physically isoluminant pure color changes as with shape changes, since color changes presumably depend primarily upon activity in the parvocellular pathway (see however, [35]), while conventional thinking has stressed the role of the magnocellular pathways in the capture of attention. However, because we only set our stimuli to physical isoluminance rather than setting them to isoluminance for each subject individually, this outcome must be regarded with considerable caution. It does suggest, however, that a more rigorous investigation of the comparative ability of color and shape to capture attention would be worthwhile.

Investigations of the effectiveness of spatial pre-cues as a joint function of cue-target SOAs and cue-target distance suggest that movements of attention occur at a velocity of approximately 8 ms/degree [34]. Assuming a movement velocity of 8 ms/deg., it would take a minimum of 64 ms to shift attention from fixation to the critical (new) item, given an eccentricity of 8.0° and the unlikely assumption of a 0 ms latency to initiate the shift. The actual time course for attention to arrive at the critical location must be longer than this, because detecting a change and initiating a shift of attention are both time-consuming processes. These considerations suggest that when the duration of the new (Frame 2) item was 50 ms, attention arrived at the critical location *after* the new item had disappeared. In this case, identification of the new item would depend upon attentive processing of a persisting but rapidly fading representation, i.e., attentive processing of the visual icon [1], [3], [33]. As the duration of Frame 2 increases to 100 or 500 ms, the likelihood increases that attention will arrive at the critical location while the new item is still present or its icon persists, resulting in a corresponding increase in identification performance.

In summary, the results of Experiment 1 strongly support the hypothesis that change detection and change identification are mediated by distinct mechanisms, with detection and localization occurring pre-attentively. In contrast, identification (of both Frame 1 and Frame 2) of the object that changed appears to be mediated largely by processes that depend upon attention. However, in contrast to some investigators (e.g. [31], [32], [36]–[38]), we will argue below that attentive processing is not the only route by which the identity of complex stimuli can be encoded.

#### Evidence of partial information concerning the type of change

Identification performance of the critical Frame 1 element, while poor, was significantly better than chance. One way to account for this above chance performance would be to attribute it to the identification and storage of a small subset of the Frame 1 items. We address this possibility later in this paper. An explanation of this kind does not, however, account for the unexpected observation that the probability of a correct Frame 1 identification depended on the *type of change* that had occurred: Frame 1 identification accuracy rates were lower when an element's color and shape changed together than when only the shape or color changed. Note that this pattern is quite different than the pattern found for change detection, where color changes were detected at the lowest rate (see *Change Detection and Localization Accuracy*). Our analysis of the trials in which the Frame 1 element was *incorrectly* identified suggests this outcome occurred because subjects had partial knowledge of the *type of change*, and could use this knowledge (in combination with knowledge of the Frame 2 item identity) to limit their guessing options. However, this information was obscured when the color and shape changed together. In this case, the combined changes in color and shape were confused with changes in shape alone. The fact that the Frame 1 identification rates depended upon correct identification of the Frame 2 item is consistent with this hypothesis.

This confusion between shape-only and combined shape-color changes can potentially be explained at more than one processing level. It is well established that early retinal processing begins a process of segregating information between different parallel channels that ultimately become specialized for different aspects of scene analysis. One would expect that changes in color would be processed primarily by parvocellular channels, whereas changes in shape would be dominantly processed by the magnocellular channels that stimulate motion-sensitive mechanisms [39]. When color and shape changes occurred in tandem, one could argue that the parvocellular neural signals that encoded the color changes could be suppressed by magnocellular signals that encoded the shape-changes [40], [41]. Alternatively, it is possible that local motion signals generated by the shape transformations were sufficiently salient to overwhelm and mask co-occurring color-change information at a more perceptual level [24], [42]. Informal observations give some credence to this interpretation, since motion percepts are sometimes evident during shape changes. An alternate explanation can be framed in terms of attentional processes. It has been argued that attention to one type of sensory attribute suppresses attention to other types of sensory attributes [43]–[47]. If color and shape changes compete for attention and the shape changes tend to dominate, the shape-only and combined color-shape changes may appear very similar. These different accounts are not mutually exclusive and our data do not allow us to discriminate between them, as all three predict the pattern of the data presented in Table 1.

## Experiment 2

### Introduction

Experiment 1 suggests that a critical factor limiting an observer's ability to encode the identity of the critical element in Frame 1 is an absence of attention to that element prior to the change. The results are also consistent with the idea that detection of the change serves to draw attention to the critical location, which facilitates encoding of the new (Frame 2) element. This new element presumably overwrites any persistence of the representation of the prior (Frame 1) item, so a shift of attention produced by the change cannot provide any information about the Frame 1 item's identity.

Visual changes are not restricted to the replacement of an old item by a new item, however. Isolated onsets or offsets also signify change, and both are efficient attractors of visual attention (e.g. [48], [49]). Many neurons throughout the central visual pathways produce robust responses to visual offsets (e.g., [9], [50], [51]), and there are circumstances in which observers detect the offset of a stimulus without having detected its onset [52]. The offset of an item should therefore be sufficient to draw attention to that location, where (in the absence of a masking stimulus) a representation should persist beyond that offset (the icon: [33], [53]–[55]). One would therefore expect substantially better recall of a critical item that is deleted rather than being replaced by a new item. Experiment 2 evaluates the magnitude of this expected improvement.

The ability of observers to identity Frame 1 items in Experiment 1 could have been mediated not only by information regarding the type of change that had occurred but also by the actual encoding of some item identities prior to the change. For instance, observers might have been able to direct their attention to a small subset of the Frame 1 items during the 500 ms presentation and store those items in short-term visual memory. If items were stored prior to the offset of the critical stimulus, an improvement in Frame 1 identification performance would be expected as the number of items presented is decreased. This is because as the number of items is reduced, the likelihood that an item stored happens to be the one that changes will increase. Experiment 2 evaluated this possibility by varying the number of items displayed in the ring of stimuli. While numerous previous reports have documented declines in performance in VSTM tasks with increases in set size using non-alphanumeric stimuli (e.g., [56]–[58]), these reports have generally measured performance in a change detection rather than change identification task. We were particularly interested in the retention of item identity information, especially with stimuli (such as ours) where accurate identifications require the encoding of conjoined shape and color information.

### Methods

#### Stimuli and procedures

Stimuli were generated and presented using the same software and equipment used in Experiment 1. In this experiment however, the Frame 1 display consisted of 3, 6, 12 or 18 elements around a circle of radius 8°. In the 18-element condition all the locations were occupied and the elements were equally spaced (as in Experiment 1). In the 3, 6, and 12 element conditions, the positions were randomly selected from the set of 18 possible locations with the same constraints on color/shape repetitions as Experiment 1. Frame 2 was identical to Frame 1 but one item (the ‘critical’ element) was deleted. Frame 1 was presented for 500 ms and then replaced by Frame 2 for either 100 ms or 500 ms (the 50 ms duration was eliminated because the asynchrony between the offset of the critical item and the remaining display items was too difficult to detect at 50 ms). After a fixed delay of 1500 ms from the onset of Frame 1, a location-response display like that used in Experiment 1 was presented. The observers indicated their detection and localization in the same manner as Experiment 1. If a change was reported, only a single set of color options and shape options was needed in the report identity display. When participants had made their selections the identification-display was replaced with a central fixation spot. Fifteen hundred ms later, the subject could initiate the next trial.

Each experimental run included 10 trials for each combination of set-size (3, 6, 12 or 18 items) and Frame 2 duration (100 or 500 ms). There were an additional 20 catch trials that were evenly distributed across the four set- sizes. An experimental run therefore consisted of 100 trials that were presented in a pseudorandom order. As in Experiment 1, observers were instructed to maintain central fixation throughout the duration of the multi-item display and the accuracy of their fixations were monitored using the same procedures employed in Experiment 1.

#### Participants

Six observers (three of whom participated in Experiment 1, including two of the authors) with normal or corrected to normal vision participated in this experiment (4 females and 2 males). Five observers participated in 10 experimental runs, and one observer in 8 experimental runs.

### Results

A total of 5800 trials were evaluated: 1000 each for five of the six participants, and 800 for the 6^th^ subject. We rejected 2.96% of those trials because of breaks in fixation and an additional 3.22% because of eyetracker malfunctions (e.g., eye blinks, head movements) leaving 93.82% of the trials (5442) available for the data analysis. Frame 2 duration had no effect on any dependent measure so the reported results are based on data collapsed across the short (100 ms) and long (500 ms) Frame 2 durations.

Averaged across the six observers, the mean hit rate for detecting a change (the disappearance of an element in frame 2) was nearly perfect (99.7%) while the false alarm rate was extremely low (0.5% overall). As in Experiment 1, participants were also very accurate in reporting the location of the change. The location was correctly reported on 91.4% of the trials, and location errors were never off by more than 1 position.

**Figure 4:**
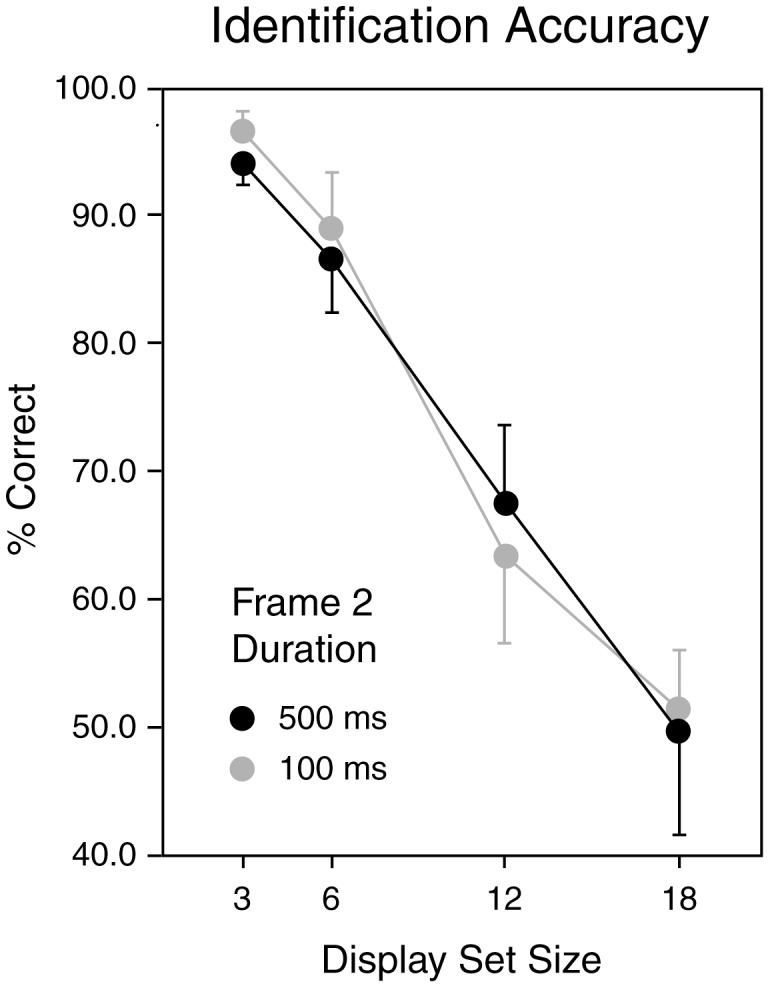
Identification accuracy in **Experiment 2.** Accuracy rates (averaged across observers) are plotted as a function of stimulus set size (3, 6, 12 and 18 items) and exposure duration (100 or 500 ms). Error bars indicate 1 SEM.

As expected, observers' performance at identifying the Frame 1 element was significantly better than in Experiment 1. Using the comparable 18-item displays, the overall correct identification rate increased from 18.5% in Experiment 1 to 50.58% in Experiment 2 (*t*(15) = 3.67, *p*<0.003). In addition, there was considerable further improvement as the number of items displayed was reduced. Figure 4 presents the percentage of correct identifications as a function of the number of items displayed. A one-way repeated measures ANOVA confirms that the number of items had a highly significant effect on identification accuracy (*F*(3,15) = 42.8, *p*<0.000001).

**Figure 5:**
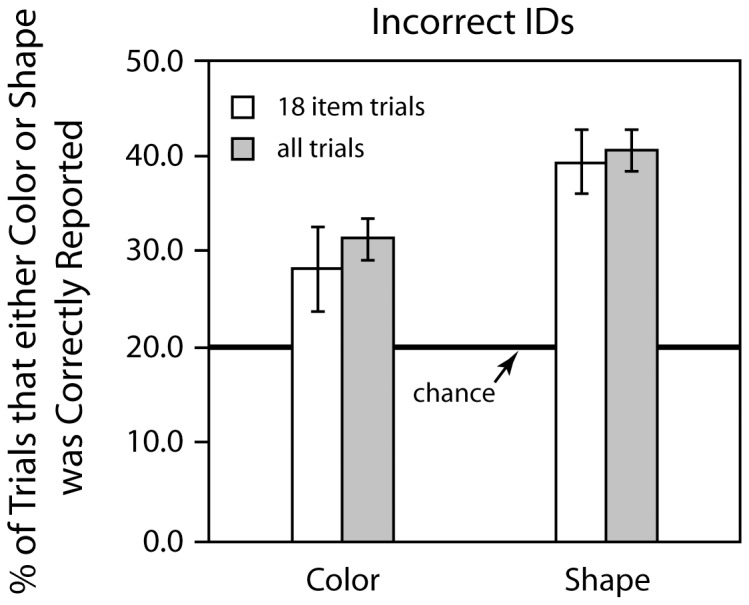
The percent correct color or shape identifications on error trials in **Experiment 2.** These data represent the percentage of correct shape or color reports for those trials in which the deleted item was misidentified, and compares the obtained results to those expected by random guessing. Error bars indicated +/−1 SEM.

In additional analyses we considered only the trials in which an identification error occurred. We determined the percentage of these trials in which either the color or shape of the item that had vanished was correctly identified although the exact item was not correctly specified, and compared these values with the 20% (3/15) rate one would expect if observers were making a completely random selection (15 items in the set of possible choices because the correct item was not an allowable choice: of those 15 items, 3 have the correct color and 3 have the correct shape). This was done for both the 18-item condition and the combined data from all the conditions. Results are presented in Figure 5.

Performance was well above chance. When the complete set of error trials is considered, this above chance performance is statistically reliable for both color and shape (*t*(5) = 5.701, *p*<0.005 for color, *t*(5) = 9.3846, *p* = 0.0005 for shape). When only the 18-item trials are considered, the above chance performance is significant for shape (*t*(5) = 6.1191, *p*<0.002), but falls slightly short of conventional significance for color (*t*(5) = 2.1932, *p*<0.08). However, when there was a color-only change, every subject reported a color-only change at better than the 20% chance rate. Finally, if one considers the entire data set, participants were less likely to make a shape error than a color error (*t*(5) = 3.86 *p*<0.02), although this difference was not significant if only the 18-item condition is considered (*t*(5) = 1.96, *p*>0.1, 2-tailed). Overall, in accord with Experiment 1, the data indicates that even when participants incorrectly identified the Frame 1 stimulus, they sometimes had partial information regarding the features of the deleted object.

### Discussion: Experiment 2

There are two principle findings from Experiment 2. First, identification accuracy is substantially improved when an item is simply deleted rather than replaced with a new item. This is evident when the results from Experiment 1 are compared to the comparable 18-item condition of Experiment 2. Second, as the number of items in Experiment 2 was reduced, there was a considerable additional improvement in performance. We will consider each of these outcomes in turn.

#### Identification accuracy: attention to the icon

It is well established that both neural responses and phenomenological experience last longer than the physical duration of a visual stimulus [9], [53]–[55], [59]. A persisting representation (e.g., the ‘icon’) can provide information about an item's identity after the item has disappeared (e.g., [33]). The results of Experiment 2 suggest that the offset of a display element generates a neural ‘offset’ response that captures attention and enables attentional processing of the icon. Note this explanation requires a transient offset response that occurs concurrently with a persisting signal that maintains the icon. This co-occurrence does not constitute a contradiction. There is both physiological evidence (e.g., [60], [61]) and psychophysical evidence ([52], [62]) that ‘on’ and ‘off’ mechanisms remain parallel independent information channels at least into the early stages of cortical processing, including the site of binocular fusion [62]. There is no reason to assume that ‘off responses’ cannot be accompanied by persisting ‘on responses’. Indeed, the capture of attention by a transient short latency off response would be of no benefit if there were not also a persisting representation on which attention could operate.

In both Experiments 1 and 2, an isolated spatio-temporal transient captured attention and this enabled improved identification accuracy at the location following the transient. In both Experiment 2 and in the 50 ms Frame 2 condition of Experiment 1, attention had to operate on the icon of the critical element rather than the image of the item itself. In Experiment 1, however, the presentation of the Frame 2 display provided an extra 50 ms during which attention could shift to the critical location before the Frame 2 offset. As a result, the persisting representation of the critical item should have been stronger in Experiment 1 than in Experiment 2, allowing the icon to produce a greater benefit. This is in accord with our observations: averaged across the change types, the accuracy rates for the Frame 2 judgments in the 50 ms condition of Experiment 1 were about 13% higher (63% vs. 50%) than the judgments for the critical element in the comparable 18-item conditions of Experiment 2 (although, due to heterogeneity of variance, this difference does not reach conventional significance, *t*(5.9) = 1.92, *p* = 0.105). This was the case despite the higher memory load in Experiment 1, in which participants needed to report the color and shape of two elements as opposed to only one in Experiment 2.

If we accept the premise that the superior performance in Experiment 1 is attributable to attending to a clearer icon, and make a simplifying assumption that the icon decays as a linear function of time, we can estimate the slope of the iconic decay function as 13%/50 ms. At this rate of decay, the overall duration of the persistence would be 385 ms, which is a reasonable estimate of the duration of iconic memory [63]–[66]. We note, however, that this estimate may be inflated because it includes any cost associated with the higher memory load imposed by the task in Experiment 1. We also acknowledge that the decay function of the icon is probably exponential. However, the decay is approximately linear during its first 300 ms, and it is in this initial period that the icon is most useful [67].

The utilization of persisting information has also been demonstrated [1], [68] in the flicker paradigm by presenting spatial cues during the blank interval and showing these cues can help overcome change blindness. The present finding is distinguished from these earlier studies by the fact that no external cue is employed; it is the offset of the critical item that draws attention to the location of the deletion. Moreover, in the case of the report by Landman et al. [68], it does not appear that the persisting information is the icon as it is traditionally conceived, since the cues were effective for as long as 1500 ms after the offset of the Frame 1 stimuli, far longer than any reported estimate of iconic duration. In fact, the exceptional duration of the informational persistence reported by Landman et al. [68] raises a question as to whether it was visual in nature. Whether or not this was the case, these findings highlight the need for care when attempting to generalize findings from change blindness to other situations in which change detection is operating efficiently.

#### Identification accuracy: Number of Items displayed

Turning to the effect of number of items in the display, we previously noted that if a small set of items was stored in VSTM during the Frame 1 presentations, performance would increase as the Frame 1 stimulus set size was reduced. This is because the odds that the critical item would be included in the set of encoded items would improve as the set size is reduced. This is what we observed. When only three items were presented, performance was almost perfect. If no items were stored in short-term visual memory prior to the change, then the number of items in the array would not be expected to have any effect: Observers would simply wait until the critical item disappeared, then direct their attention to the icon at that location. The fact that identification accuracy increased dramatically as the number of items decreased therefore implies that some of the items are identified *before* the location of the critical element is known. This might occur if participants covertly shifted their attention from item to item despite maintaining central fixation. In this case, the number of items that can be stored would be limited both by the capacity of visual working memory and by the rate that attention can switch. Estimates of this rate show a great deal of method-dependent variability. The slope of search functions can be less than 50 ms/item (e.g. [2], [38], [69]), but in a paradigm that specifically required subjects to attend to two successive locations in a set of simultaneously presented targets, the minimum ‘dwell time’ of attention was estimated to be about 250 ms [70]. Most studies show even longer dwell times: estimates based on the ‘attentional blink’ [71], attention shifts between rapid serial visual presentation streams [72] and inhibition of return in visual identification tasks [73] all indicate that, once engaged, it can take as long as 500 ms to shift attention to a new location. Given the uncertainty regarding the time required to switch spatial attention, a limited amount of switching between Frame 1 targets cannot be discounted as a possibility. Another possibility is that there is a spread of the focus of attention so that it encompassed multiple items in the Frame 1 display [74]. In this case, one might expect items close to the critical item to derive more benefit than items farther away. A third way of accounting for our data would be the parallel encoding of a small number of element identities [75], [76] despite the absence of directed spatial attention (Lachter et al. [37] notwithstanding). In this case, in addition to the capacity of working memory, the number of items stored might be determined by limits in the speed of the parallel encoding process. In Experiment 3 we used a visual cueing paradigm to try and differentiate between the attention switching, spread of attention and parallel processing accounts.

## Experiment 3

### Introduction

In Experiment 3, we attempted to control the status of attention before the change by providing spatial pre-cues that predicted the location of the upcoming change. We expected that observers would direct their attention to the cued location, leaving the remaining locations unattended. Although we had no way of evaluating the spatial spread of attention prior to performing this experiment, the pattern of results we obtained on invalid trials suggests it was quite sharply focused on the cued item. Experiment 2 suggested that a small number of element identities are stored in VSTM before any change occurs. We reasoned that if this was due to sequential shifts of attention, anchoring attention in this manner would impair or block this storage process. On the other hand, if storage was the result of a parallel process that did not depend upon focused attention, anchoring attention should have little or no effect.

### Methods

Eights observers, including two of the authors and six Dartmouth undergraduates, participated in Experiment 3. Two of the undergraduates had participated in Experiment 1, and one had participated in Experiment 2.

The general experimental procedures were similar to those in Experiment 1: an element changed either color or shape in two successive frames. In order to reduce the number of trial types required (see Table 2), we eliminated the condition in which both color and shape could change. Eye position was recorded as before to insure accurate fixation using the same methods used in Experiments 1 and 2. After fixating a central point, participants initiated each trial with a mouse click. The display sequence was similar to that used in Experiment 1, with the following modifications: the maximum number of potential display positions in the ring of items was reduced from 18 to 12, distributed as hourly clock positions. Prior to the presentation of Frame 1, a predictive pre-cue (80% valid) was presented just (0.8°) interior to one position on the ring of display locations to indicate where the change was most likely to occur. The cue was a small (0.25°) white dot that flashed (with a 50% duty cycle) at 10 Hz for 500 ms. Three hundred ms after the cue presentation, the Frame 1 display appeared for 500 ms and was followed immediately by the Frame 2 display for 500 ms. The sequence of response screens used in Experiment 1 was then presented.

**Table 2:** This table presents the frequency of the different types of trials in Experiment 3 (84 trials/experimental run).

Valid 3 items	Valid 12 items	InvalidNear 3 items	InvalidNear 12 items	InvalidFar 3 items	InvalidFar 12 items	Neutral 3 items	Neutral12 items	Catch3 items	Catch 12 items	NeutralCatch 3 items	NeutralCatch 12 items
24 trials	24 trials	4 trials	4 trials	4 trials	4 trials	8 trials	8 trials	8 trials	8 trials	2 trials	2 trials

There were 84 trials in an experimental run, divided into 12 display conditions, as described below. Half the trials presented a 3-item display and half presented a 12-item display. On *valid trials* the change occurred at the cued location. On *invalid trials* the change occurred at an uncued location. The invalid trials were divided into *invalid-near* trials in which the change occurred either one or two clockwise or counterclockwise steps from the cued location and *invalid-far* trials in which the change occurred on the side of the display opposite the cued location, no more than two steps (clockwise or counterclockwise) from the antipode of that location. To allow adherence to this scheme, when three items were presented, they were always displayed at the cued location, an un-cued far location and an un-cued near location. In addition, there were a series of neutral trials in which the pre-cue was presented overlapping the central fixation point rather than peripherally, and a set of catch trials in which no change ever occurred. Table 2 presents the twelve display conditions and their distribution within each experimental run.

While elaborate, this set of conditions allows an evaluation of both the benefits of directing attention to the location that would change and the costs associated with misdirecting attention as a function of spatial distance. We reasoned that using the spatial pre-cue should anchor attention and prevent any sequential deployment of attention to multiple items during the Frame 1 presentation. If the benefit in Experiment 2 produced by reductions in the number of Frame 1 items is largely unaffected by the pre-cues, it would argue that this benefit is based on parallel (simultaneous) processing of the Frame 1 items rather than sequential shifts of visual attention during Frame 1.

### Results

#### Hits, false alarms, and Location accuracy

As in the Experiments 1 and 2, observers' detection and localization performance were essentially errorless. Across observers and conditions, hits occurred on 99. 9% of the change trials and false-alarms occurred on only 0.7% of the catch trials. Overall, the change was correctly localized on more than 99% of the trials, and there were no location errors more than one position away from the actual change. Because hit rates were near ceiling and localization errors virtually absent irrespective of the cuing condition, there was no indication that misdirecting attention had a negative impact on subject's change detection and localization abilities. This was the case for both the 3-item and 12-item displays. A meaningful statistical comparison of detection rates as a function of change type was not viable because these rates were near ceiling in every change type condition.

#### Identification Performance on Frame 1

**Figure 6:**
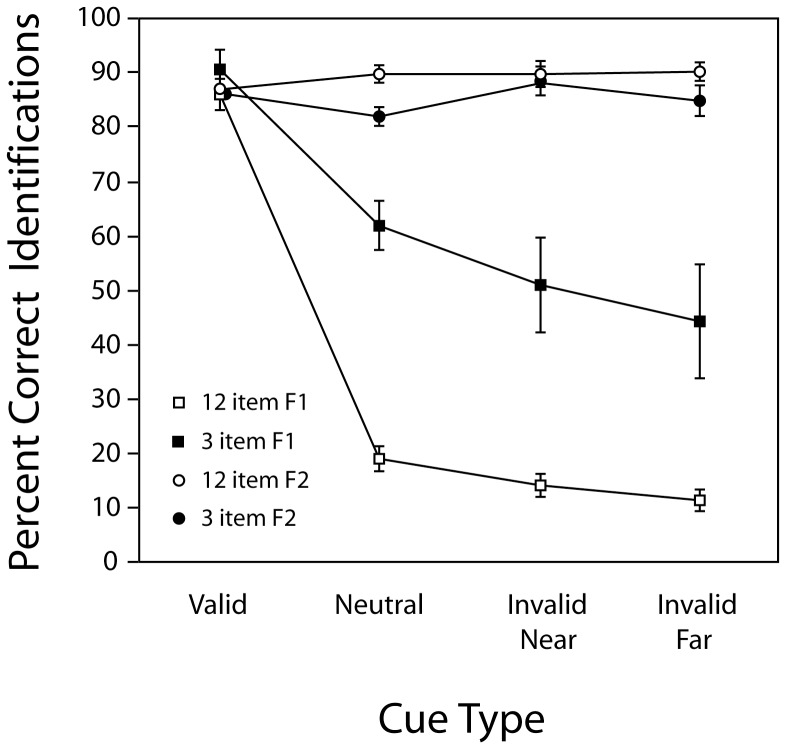
The percentage of correct identifications for each cuing condition in **Experiment 3.** Averaged accuracy rates for the critical item in Frame 1 (squares) and Frame 2 (circles) are illustrated for 12 item (open symbol) and 3 item (filled symbol) displays as function of the cuing conditions (valid, neutral, invalid near and invalid far). Error bars indicate +/−1 SEM. See text for details.

Frame 1 and Frame 2 identification accuracy rates in all the cuing conditions for both the 3-item and 12-item displays are shown in Figure 6. The accuracy data for Frame 1 was first analyzed by a 2-way repeated measures ANOVA with four levels of the factor Cue-Type (neutral, valid, invalid near, invalid far) and two levels of the factor Number-of-Items (12 and 3). This analysis showed a significant effect for both factors (*F*(3,21) = 70.5 , *p*<0.000001 for cue type, *F*(3,21) = 35.6, *p*<0.001 for number of items) and a significant interaction between them (*F*(3,21) = 18.2, *p*<0.00001). Following this analysis, 2 tailed post-hoc t-tests were used to compare specific condition means.

When the experimental conditions were comparable, the results from Experiment 3 closely replicate those in Experiments 1 and 2. For example, on *neutral trials*, identification accuracy with 12-item displays was slightly higher than that observed using 18-item displays in Experiment 1. Accuracy rates were much better when three items were presented than when 12 items were presented, as was the case in Experiment 2. In Experiment 3, participants correctly identified the Frame 1 stimuli on 61.8% of the 3-item trials and on 18.9% of the 12-item trials. This difference was highly significant (*t*(7) = 12.63, *p*<0.0001). However, even with the 12-item display the accuracy rate is statistically better than the expected chance level of 1/15 (*t*(7) = 5.93, *p*<0.001).

In the *valid cue* conditions, observers correctly identified the Frame 1 element on 87.1% of the trials with the 12-item display, and 86.4% of the trials with the 3-item display. These accuracy rates are significantly better than those in the corresponding neutral cue conditions (*t*(7) = 14.35, *p*<0.0001 for 12 items, *t*(7) = 6.52 *p*<0.001 for three items) and do not differ from each other (*p*>0.1).

When the cue was *invalid*, an effect of the number of items presented was again apparent. With a 3-item display, Frame 1 identifications were correct on 44.3% of the trials with an *invalid-far cue*, and 51% of the trials with an *invalid near cue*. With a 12-item display, the corresponding accuracy rates are 11.6% and 14.1%. Thus, the change from 3 to 12 items produced a decline in performance of 32.7% with the invalid-far cue and 36.9% with the invalid-near cue. These decrements are not significantly different from each other, and not significantly different from the comparable decline found in the neutral cue condition (*p*>0.1 for all comparisons). While accuracy rates on the invalid trials are lower than those observed on neutral trials, it is only in the case of the invalid-far trials that this difference reaches 2-tailed significance (*p*<0.03 with the 3-item display, *p*<0.005 with the 12-item display). However, the accuracy rates in the invalid-near and invalid–far conditions are not significantly different.

#### The Spatial Gradient of Attention

**Figure 7:**
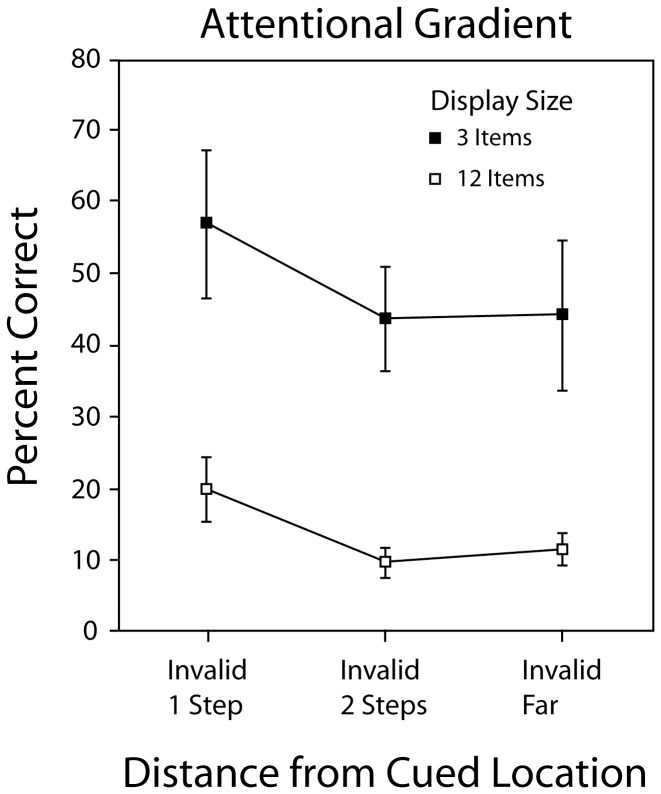
Frame 1 Identification accuracy in **Experiment 3** **on Invalid trials.** Accuracy is a function of distance between the cued location and the location of the change. The gradient of attention is the same for the 3 and 12 item displays.

In an additional analysis we searched for a finer-grained spatial gradient of attention in the near-invalid condition (cue invalid by one position vs. cue invalid by two positions). When 12 items were presented, the accuracy rate was 19.8% when the target was displaced from the cue by one step, and fell to 9.5% when it was displaced from the cued position by two steps (*t*(7) = 2.3, *p* = 0.055). When three items were presented, the accuracy rate was 57% when the target was displaced from the cue by one step and fell to 43.6% when it was displaced by two steps (*t*(7) = 2.73, *p*<0.03). The accuracy rates obtained when the change occurred 2 steps from the cue are no better than those found in the invalid-far condition (see Figure 7). The data therefore reveal a very steep gradient of attention for both the 3 and 12-item displays: in each case a miss was only better than a mile if it was a very near miss.

#### Identification Performance on Frame 2

Identification accuracy rates for Frame 2 were high irrespective of the cuing condition (see Figure 6). A 2-way repeated measures ANOVA with cue-type and number-of-items as factors indicated that the main effect of cue type was non-significant (*F*(3,21) = 1.6, *p* = 0.22), the main effect of number-of-items was significant (*F*(1,7) = 6.46, *p*<0.04), and the interaction between these factors falls just short of significance (*F*(3,21) = 2.67, *p*<0.08). The absence of a cuing effect in Frame 2 is not surprising if one accepts the proposition that the change captures attention. The main effect of number-of-elements on Frame 2 accuracy reflects slightly poorer overall performance with the 3 element displays (85%) than with the 12 element displays (89%). We cannot account for this outcome, and despite its statistical significance we suspect it reflects only a chance fluctuation in the data.

### Discussion: Experiment 3

Experiment 3 demonstrates that both spatial pre-cues and the number of items displayed have strong and interacting effects on Frame 1 identification accuracy. When the cue was valid, the identification accuracy for the critical item in Frame 1 was very high, demonstrating that participants were attending to the cued location, and confirming that the poor Frame 1 performance in the previous experiments was related to an absence of focused attention on the critical item prior to any change. When the cue was invalid, we assume that attention was also directed to the cued location and remained there until the change occurred. Given this assumption, the premise that attention is *necessary* for the identification of the critical Frame 1 item predicts that performance should be at chance (1/15, or 6.7%, assuming the Frame 2 item was correctly identified). Frame 1 accuracy on invalid trials averaged just over 10% for 12-item displays (see figure 6), which is slightly greater than chance. This above-chance performance might be explained if observers had some sense of the *type* of change that occurred and used this information to narrow their range of guessing options (see Experiment 1). However, identification accuracy on the invalid trials with three items increased to 43%, which is far better than chance. This high accuracy rate and the interaction between the effects of pre-cues and the number of displayed items are fundamental outcomes that need to be explained.

#### A spread of attention or attention switching?

An explanation based on the spread of attention to encompass more than one item does not seem consistent with the data shown in Figure 7, which reveals a narrow focus of attention on the cued item that is very similar for both the 3 item and the 12 item displays. An alternative explanation based on attention switching (as described in the discussion of Experiment 2) might be viable in the neutral cue condition where the focus of attention was not explicitly constrained, although if the attention dwell time is in the range of 400–500 ms [71]–[73], this would leave scant time for switching to occur during the 500 ms Frame 1 presentation. If observers switched their attention at a fixed rate irrespective of the number of items presented, a performance advantage for 3-item displays would be expected, since the proportion of displayed items that receive attention would increase as display size decreases. We think, however, that attention switching in the trials with a peripheral pre-cue is extremely unlikely. Observers knew that the cue indicated where the change was likely to occur and the excellent identification performance on valid trials confirms they were attending to that location. We cannot conclusively exclude the possibility that observers attended to the cued item in Frame 1 and encoded its identity, then *re*-directed their attention to another Frame 1 item, and encoded its identity. It seems implausible, however, that an observer would deliberately shift their attention away from the most likely location of the impending change, even if they could. Given the steepness of the observed spatial gradients, it is likely that attention was focused at the cued location at the moment the change occurred. Thus, the data from invalid trials implies that *critical items were sometimes identified prior to the change without the benefit of attentive processing.*

#### The ‘Two-Items Encoded’ Hypothesis

**Figure 8:**
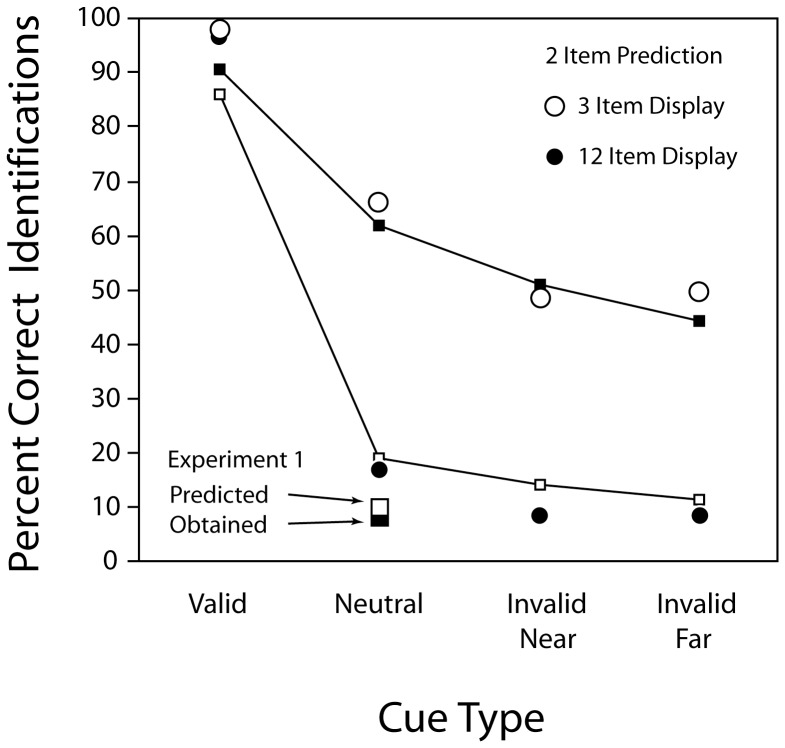
Obtained Frame 1 accuracy rates compared to predictions from the 2-items encoded hypothesis. Predictions are compared with the data from Experiment 1 (large squares) and those in Experiment 3 (small squares) for all cuing conditions and display sizes.

When we assessed this implication, we found a pattern in the data illustrated in Figure 6 that we had not anticipated: all of the Frame 1 identification data in Figure 6 can be accounted for by making the single assumption that observers were able to encode, on average, the identity of two Frame 1 items regardless of the display size or cuing condition. For instance, on neutral trials with three items this ‘two items encoded hypothesis’ predicts an accuracy rate of 67% (two items identified/three items presented), and for neutral trials with 12 items the prediction is 16.7% (two items identified/12 items presented). The accuracy rates observed closely approximate these predictions (neutral 3-item = 61.8%, neutral 12-item = 18.9%). The data from invalid trials are predicted in a similar manner if we make the additional assumption that the function of attention is simply to specify one of the two encoded items. Thus, we propose that the attended (i.e., cued) item and one additional item at a randomly selected and unattended location were identified. When three items were presented, the predicted accuracy rate on an invalid-cue trial would then be 50%. In fact, the observed accuracy rate in the 3-item invalid trails averaged 47.6%. With a 12-item invalid-cue display, there were 11 items outside the focus of attention, so the predicted accuracy rate for the critical item would be 1/11 (9.1%). The empirically obtained average Frame 1 accuracy rate in the invalid 12-item conditions was 12.9%. Figure 8 illustrates the close correspondence between the empirical results in both Experiments 1 and 3 and those predicted by the 2 items encoded hypothesis.

## Review and General Discussion

### Detection and Localization

The present data clearly indicate that the detection and localization of discrete changes do not require attention. This conclusion is based on the consistent finding in all our experiments that change detection and localization were essentially perfect regardless of the number of items in the display, and that misdirecting attention had no effect on change detection or localization. Pre-attentive change detection is consistent with previously reported results [1], [3], [23]. The fact that color and shape changes both appear to be detected pre-attentively supports the view that an omnibus change detection system is sensitive to the outputs of a very diverse array of low-level detectors (e.g., involving both magnocellular *and* parvocellular contributions).

### Detection versus Identification

In contrast to detection and localization, the results support the widely accepted view that the ability to identify stimuli undergoing change is substantially facilitated by attention. Thus change detection and change identification are fundamentally different processes [3], [17], [23], [24]. The results also support the conclusion that a discrete, isolated change captures attention. In Experiments 1 and 3, because the change draws attention to the new item, that item is usually identified, while the difficulty in identifying the critical item prior to the change is presumably due to the low likelihood of its being attended during its pre-change presentation.

The fact that detection and identification are frequently conflated (e.g., [2], [6], [13], [14], [22]) can be attributed to the difficulty in distinguishing them with commonly used change blindness paradigms such as the ‘flicker paradigm’. In the present study there was only one frame transition, and abundant instances where the change was detected and localized but the item that had changed could not be specified. In the flicker paradigm, the repetitive presentation sequence always permits detection to be immediately followed by identification. Even if identification is not possible during the frame when detection occurs, it becomes possible as soon as the next frame is presented. Because identification can occur during the frame that immediately follows change detection, it is not phenomenologically apparent that the two processes can occur in rapid succession.

Another possibility (suggested by an astute reviewer) is that the flicker paradigm suppresses the ambient change detecting systems so completely that observers are compelled to engage in an explicit comparison of items before and after the change. In this case detection, localization and identification could occur concurrently. Indeed, if a meticulous comparison of pre- and post-change items is required in change blindness paradigms, it could be argued that in these paradigms change *identification* supports change *detection*. However, the present findings clearly indicate that this is not the way scene changes are typically processed: detection normally occurs pre-attentively and facilitates identification by capturing attention.

### Object Deletions and Attention

The present account also applies when the change is the deletion of an item. In this case however, the deletion attracts attention to a location that only contains a rapidly fading icon of the deleted stimulus. Any benefit of attention capture by offsets logically requires both a short-latency offset response (that captures attention) and a parallel persisting ‘on’ response (that mediates identification of the deleted item). There is ample physiological evidence of parallel ‘on’ and ‘off’ pathways (e.g., [60], [61]) as well as evidence for the persistence of ‘on’ responses beyond the stimulus offset (e.g., [9], [59]).

### Identification without Attention

Although generally poor, Frame 1 identification accuracy was reliably better than chance. An analysis of the identification errors in Experiment 1 suggests that one factor contributing to this above chance performance may have been that observers had some information about the nature of the change (whether it entailed a change in color or shape) and that this partial information, when combined with an accurate identification of the new item (mediated by attention), improved the accuracy of guessing.

However, the results of Experiments 2 and 3 suggest that the major factor mediating the above chance performance with Frame 1 items was the encoding and storage of item identities prior to the change. In Experiment 2, if identification were entirely dependent upon the extraction of information from the icon, we would expect identification performance to be independent of the number of items in the display. Contrary to this prediction, reducing the number of items shown dramatically improved the rate of accurate Frame 1 identifications from just over 50% (when 18 items were shown) to better than 95% (when three items were shown). This can be explained if one posits that observers were able to encode and store a small number of Frame 1 item identities prior to the deletion of the critical item. If the number of items shown is much larger than the number that can be stored, the odds that the critical item is one of those that had been stored will be small, but those odds get better as the number of items in the display is reduced. This raises the question of whether the identification of items prior to the change also requires attention.

In principle, encoding items prior to any change could depend on shifting attention to a small number of items during the Frame 1 presentation. However, the results of our third experiment argue against this hypothesis. In this experiment, we employed a visual pre-cue to attract attention to a particular item in the Frame 1 array. When the cued item was the critical item, observers were able to specify the change almost 90% of the time, demonstrating that observers were in fact attending to the cued location. If it is assumed that 1) only attended items can be identified and 2) invalid peripheral precues attracted and held attention at the cued location as effectively as valid cues, then it would follow that, regardless of the number of items in the display, invalid cues should have eliminated any possibility of identifying the critical item in Frame 1 (because attention is misdirected and by assumption #1 only attended items can be identified). This, however, was clearly not the case: Frame 1 identification performance on invalid trials was far better with 3-item than 12-item displays. This outcome implies that some unattended items were identified and stored, despite the fact that this identification required the encoding of a conjunction of features. Any interpretation of the Frame 1 identification data in terms of an increased spread of attention to encompass multiple items also does not seem viable, since the spread of attention was narrow and had similar gradients with the 3 and 12-item displays (see Figure 7).

### The Two-items Encoded Hypothesis

Finally, we noted that the entire range of pre-change identification accuracies observed in Experiment 3 (from over 90% to less than 10%) could be accounted for by making one simple assumption: regardless of the focus of attention, observers were able to encode two items in these multi-element displays. This hypothesis is consistent with the data from valid, neutral and invalid pre-cue conditions in Experiment 3, and with the Frame 1 identification accuracies reported in Experiment 1 (see Figure 8). According to this view, cueing one of the items prior to the change simply insures that the attended item will be one of the two items encoded, leaving the second item outside the focus of attention (by up to 16°).

### The capacity of VSTM

We recognize that studies on the span of apprehension have reported that the capacity of short-term visual memory is approximately 4 letters, numerals, simple features or complex objects [33], [56], [69], [77], [78]. In our displays, if shape and color are considered features, then identification of two objects would be equivalent to identifying 4 features, but we also acknowledge that the capacity of VSTM has recently been construed in terms of the storage of objects versus features (e.g., [56]–[58], [79], [80]). There is evidence that storage capacity depends on the nature of the stored items [58], so it is possible that the two item encoding capacity we found was to some extent dependent on our choice of colored geometric shapes as stimuli. We also note that the need to store the changed Frame 2 item as well as Frame 1 items added to the memory load imposed by our change identification tasks. In combination, the storage of two Frame 1 items and the identified Frame 2 item requires a capacity of 3 objects. It should also be borne in mind that many of the studies on the capacity of VSTM have used a change detection rather than a change identification paradigm [56], [68], so that the number of items that observers could actually identify was not determined. Finally, it should be pointed out the two item limitation that we found need not be a reflection of the capacity of VSTM *per se*. Rather, it could reflect a limitation that is inherent to the pre-attentive encoding mechanism we are proposing. That is to say, even though VSTM may be capable of holding more items, it is possible that only 2 items could be transferred to the VSTM buffer without the support of attention.

### Other Support for the Two-Item Encoding Hypothesis

It has previously been argued that the process that consolidates items into VSTM either operates on only one item at a time or operates with progressively reduced efficiency as the number of items grows larger than one [69], [81], [82]. While the two-item hypothesis is speculative, one recent study appears to support it. Mance et al. [76] have found that the accuracy with which the color of a block is encoded into VSTM is no worse when two blocks are presented simultaneously as when they are presented sequentially, although with block sets greater than 3 the sequential presentations produce superior performance. The authors conclude this indicates the rapid parallel storage of a least two items in briefly presented displays, which is in accord with our finding that the multiple item displays we employed permitted reliable encoding of two items by a process that does not require directed attention.

Clearly, more research is required to support and evaluate the two-item hypothesis, and determine how it might be reconciled with the results of previous studies. We are currently engaged in such research. Initial findings indicate that when observers are asked to report the identity of all of the items (full report) in displays identical to those used in the experiments reported here, on average they are only able to correctly report 2 items regardless of display size. These investigations are still in progress and their results are only preliminary. Nevertheless, the data presented here leads us to conclude that while attention substantially facilitates the identification of visual objects, a very limited number of unattended items can be routinely identified and stored in VSTM without the benefit of focused attention. Thus, the fact that attention virtually insures that an item *will* be identified does not mean that an unattended item *cannot* be identified.
